# Venetoclax and Homoharringtonine‐Based Therapy Exhibited a Striking Response in Refractory/Relapsed Early T‐Cell Precursor Acute Lymphoblastic Leukemia

**DOI:** 10.1002/mco2.70549

**Published:** 2025-12-12

**Authors:** Xiang Zhang, Hongsheng Zhou, Liping Mao, Yinjun Lou, Lijing Shen, Ying Lu, Zhenfang Liu, Xiuzhen Tong, Aiping Zhang, Tingbo Liu, Na Zhang, Xingnong Ye, Juying Wei, Meihong Luo, Shaoyuan Wang, Qingxian Bai, Jian Hou, Qifa Liu, Hongyan Tong, Jie Jin, Wenjuan Yu

**Affiliations:** ^1^ Department of Hematology The First Affiliated Hospital Zhejiang University School of Medicine Hangzhou Zhejiang PR China; ^2^ Department of Hematology Nanfang Hospital Southern Medical University Guangzhou Guangdong PR China; ^3^ Departments of Hematology Renji Hospital Shanghai Jiao Tong University School of Medicine Shanghai Shanghai PR China; ^4^ Department of Hematology The Affiliated People's Hospital of Ningbo University Ningbo Zhejiang PR China; ^5^ Department of Hematology The First Affiliated Hospital of Guangxi Medical University Nanning Guangxi PR China; ^6^ Department of Hematology First Affiliated Hospital of Sun Yat Sen University Guangzhou Guangdong PR China; ^7^ Department of Hematology Baoshan Hospital Shanghai University of Traditional Chinese Medicine Shanghai Shanghai PR China; ^8^ Department of Hematology Fujian Medical University Union Hospital Fuzhou Fujian PR China; ^9^ Department of Hematology the First Affiliated Hospital of Air Force Military Medical University Xi'an Shaanxi PR China

**Keywords:** early T‐cell precursor acute lymphoblastic leukemia, refractoriness, relapse, venetoclax, homoharringtonine

## Abstract

Early T‐cell precursor acute lymphoblastic leukemia (ETP‐ALL) is an aggressive subtype of T‐ALL. Once refractory or relapsed, it is associated with a poor prognosis, with a complete remission (CR) rate of 36%–46% following re‐induction therapy. Previously, we reported a synergistic effect of venetoclax (VEN) and homoharringtonine (HHT) in ETP‐ALL, which potentially elicits notable clinical responses. Herein, we investigated the efficacy and safety of the V‐HAG regimen (VEN, HHT, cytarabine, and granulocyte colony‐stimulating factor [G‐CSF]) in patients with refractory/relapsed ETP‐ALL through a prospective, multicenter, single‐arm, open‐label, phase 2 clinical trial. A total of 18 patients were enrolled, and 100% of these patients achieved CR or CR with incomplete hematological recovery (CRi) after 1 cycle of the V‐HAG regimen as re‐induction therapy. As a follow‐up, both the relapse rate and mortality rate were 33.3%. The 1‐year overall survival and relapse‐free survival were 76.7% (95% confidence interval [CI]: 53.2%‐100.0%) and 55.7% (95% CI: 28.8%–82.6%), respectively. The most common grade 3–4 adverse events were neutropenia (100%), anemia (88.9%), and thrombocytopenia (100%). Notably, the VEN‐ and HHT‐based therapy, V‐HAG regimen, exhibits an extremely high efficacy in the treatment of patients with refractory/relapsed ETP‐ALL with good tolerance, and it provides a promising therapeutic strategy for improving their outcomes.

## Graphical abstract

Early T‐cell precursor acute lymphoblastic leukemia (ETP‐ALL) was an aggressive subtype of T‐ALL with an extremely poor prognosis. We demonstrated that the V‐HAG regimen (venetoclax, homoharringtonine, cytarabine, and G‐CSF) was highly effective in refractory/relapsed patients. Eighteen patients were enrolled, and 100% of patients achieved complete remission (CR) or CR with incomplete hematological recovery after 1 cycle of the V‐HAG regimen as re‐induction, which was also well‐tolerated. The 1‐year overall survival (OS) and relapse‐free survival (RFS) were 76.7% (95% CI, 53.2%–100.0%) and 55.7% (95% CI, 28.8%–82.6%), respectively.

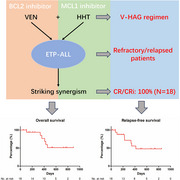

## 1 Introduction

T‐cell acute lymphoblastic leukemia (T‐ALL) is an aggressive hematological malignancy arising from immature T cells. Based on immunophenotypic and transcriptional characteristics, T‐ALL can be divided into two subtypes: early T‐cell precursor acute lymphoblastic leukemia (ETP‐ALL) and non‐ETP T‐ALL [1, 2, 3, 4]. Currently, intensive chemotherapy, such as the alternating hyper‐CVAD/MA regimen, combined with allogeneic hematopoietic stem cell transplantation (allo‐HSCT), constitutes the standard therapeutic strategy for T‐ALL [5]. Compared with non‐ETP T‐ALL, ETP‐ALL exhibits a poor response to standard chemotherapy and a high propensity for relapse, resulting in an extremely unfavorable prognosis. Once refractory or relapsed ETP‐ALL is confirmed, allo‐HSCT remains the only potential curative option for achieving long‐term survival. However, the complete remission (CR) rate following re‐induction therapy with currently available regimens ranges from 36% to 46% [6, 7, 8, 9]. Therefore, developing effective strategies to bridge patients with refractory/relapsed ETP‐ALL to allo‐HSCT represents the most critical clinical challenge in this patient population.

It is widely recognized that ETP‐ALL is an aggressive, poorly differentiated stem cell‐like leukemia that shares similar properties with hematopoietic stem cells and myeloid progenitor cells [1]. Genetically, ETP‐ALL is characterized by activating mutations in genes that regulate cytokine receptor and RAS signaling pathways, as well as inactivating lesions that disrupt hematopoietic development and histone‐modifying genes. Compared with non‐ETP T‐ALL, ETP‐ALL exhibits a higher frequency of myeloid lineage‐associated gene mutations (e.g., *FLT3*, *DNMT3A*) but a lower frequency of other T‐ALL‐associated mutations (e.g., *NOTCH1*, *CDKN1/2*) [1, 10]. Consistently, in addition to T‐ALL‐like treatment regimens, myeloid leukemia‐like chemotherapy regimens have shown potential efficacy in ETP‐ALL patients [11, 12]. Furthermore, several targeted therapeutic agents have been developed based on the intrinsic biological features of ETP‐ALL, including JAK inhibitors, hypomethylating agents (HMAs), histone deacetylase inhibitors, FLT3 inhibitors, CD33‐conjugated antibodies, and CD38 monoclonal antibodies [13]. However, genetic characteristics and immune phenotypes do not perfectly align in T‐ALL, and ETP‐ALL also exhibits significant genetic heterogeneity. Thus, there remains an unmet need for a universally effective targeted therapy for ETP‐ALL.

The BCL2 inhibitor venetoclax (VEN) has been successfully utilized in the treatment of various hematological malignancies, particularly chronic lymphocytic leukemia (CLL) and acute myeloid leukemia (AML) [14, 15]. It is widely accepted that sensitivity to VEN is primarily determined by a high BCL2/BCL2L1 ratio and a low MCL1 level. As previously reported, ETP‐ALL exhibits an earlier differentiation stage and greater proximity to hematopoietic stem/progenitor cells compared with non‐ETP T‐ALL; consequently, a high BCL2/BCL2L1 ratio is observed in ETP‐ALL but not in non‐ETP T‐ALL [16]. This finding has demonstrated that ETP‐ALL is also a hematological malignancy highly dependent on BCL2. Consistently, preclinical studies have shown that ETP‐ALL is sensitive to VEN, highlighting significant potential for its clinical application in treating this disease. The combination of VEN and HMAs is recognized as the standard therapy for elderly AML patients, and their based therapy has also been shown to be highly effective in patients with refractory/relapsed ETP‐ALL, achieving a composite CR (cCR) rate of 66.6% [17, 18]. Nevertheless, the efficacy of VEN‐based regimens in ETP‐ALL treatment could potentially be enhanced by substituting HMAs with novel therapeutic agents.

Homoharringtonine (HHT), a classic antileukemic agent in China, is originally isolated from *Cephalotaxus hainanensis* and is widely recognized as a protein synthesis inhibitor [19]. Our research group has dedicated significant efforts to investigating the efficacy and mechanism of HHT in AML, and has demonstrated that HHT‐based regimens exhibit excellent efficacy in both de novo and refractory/relapsed AML. HHT exerts its antileukemic effects primarily by downregulating FLT3 and MYC [20, 21, 22, 23]. Notably, MCL1, whose overexpression directly mediates VEN resistance, is also a therapeutic target of HHT in AML; thus, HHT has the potential to prevent and overcome VEN resistance when used in combination with VEN [24, 25]. There is no doubt that VEN‐ and HHT‐based therapies have shown outstanding efficacy in AML treatment in both preclinical and clinical studies [26]. Additionally, our team has also observed the efficacy of HHT in T‐ALL [27]. Therefore, VEN‐ and HHT‐based therapies hold promise for the treatment of ETP‐ALL.

Previously, we successfully induced CR in a patient with relapsed ETP‐ALL using a regimen consisting of VEN, HHT, cytarabine, and granulocyte colony‐stimulating factor (G‐CSF) (referred to as the V‐HAG regimen), followed by bridging to allo‐HSCT [12]. Importantly, VEN and HHT exert a synergistic inhibitory effect on ETP‐ALL, similar to their interaction in AML [28, 29, 30]. Subsequently, our pilot study demonstrated that the V‐HAG regimen, when used as re‐induction therapy for refractory/relapsed ETP‐ALL, achieves surprisingly excellent efficacy [28]. Herein, we conducted a clinical trial in adult patients with refractory/relapsed ETP‐ALL to evaluate the efficacy and safety of the V‐HAG regimen as a re‐induction therapy. Strikingly, a cCR rate of 100% was observed.

## 2 Results

### 2.1 Patient Characteristics

**FIGURE 1:**
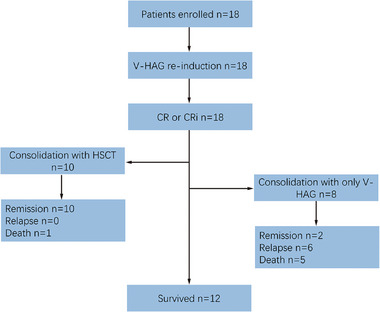
Summary of the study. As of September 31, 2024, 18 patients with refractory/relapsed ETP‐ALL were enrolled to receive V‐HAG treatment, and all achieved CR or CRi. Among these patients, 10 were bridged to HSCT, and their remission was well maintained. In contrast, most of the 8 patients who did not receive HSCT lost their remission. As of the last follow‐up date, 12 patients remained alive.

From December 1, 2021, to September 31, 2024, a total of 18 patients from 9 centers were enrolled in this study (Figure 1). The baseline characteristics of these 18 evaluable patients are summarized in Table 1. The median age was 40.5 years (range: 19–73 years), and the male‐to‐female ratio was 2:1. Among the patients, 9 were diagnosed with refractory ETP‐ALL, and 9 with relapsed ETP‐ALL. Of the refractory patients, 7 were refractory immediately after initial diagnosis, and 2 became refractory following relapse. Among the relapsed patients, 7 experienced their first relapse, and 2 experienced a second or subsequent relapse. At the time of confirmation of refractory/relapsed disease, 14 patients received the V‐HAG regimen as first‐line re‐induction therapy, while 4 patients had previously undergone other chemotherapy regimens. Notably, only Patient #1 had received a venetoclax (VEN)‐based therapy (the V‐CAG regimen: VEN, aclacinomycin, cytarabine, and G‐CSF) during their first relapse, which resulted in the achievement of a second CR. Upon confirmation of a second relapse, this patient was treated with the V‐HAG regimen. All other patients had no prior exposure to VEN. Molecular biological analysis results were available for all 18 patients, and significant genetic heterogeneity was observed among these patients. Consistent with previous reports, genes encoding epigenetic regulators were frequently mutated in ETP‐ALL; among these, *DNMT3A* mutations were the most common, with a frequency of 33.3%. Additionally, *TP53* mutations were detected in 11.1% of the patients (Table 2).

**TABLE 1:** Characteristics of enrolled refractory/relapsed ETP‐ALL patients.

Characteristics	*N* (%)
Age (years)	
Median (range)	40.5 (19–73)
Gender	
Male	12 (66.7%)
Female	6 (33.3%)
Refractory/relapsed disease	
Refractory	9 (50.0%)
Relapsed	9 (50.0%)
Hematologic parameters	
Median (range)	
White blood cell count (10^9^/L)	2.18 (0.41–9.68)
Hemoglobin level (g/L)	77.5 (50–135)
Platelet count (10^9^/L)	81 (8–408)
Bone marrow blast	
Median (range)	66.0% (8.0–95.0)
Karyotype	
Normal	14 (77.8%)
Abnormal	2 (11.1%)
Unknown	2 (11.1%)
Therapeutic line of V‐HAG for re‐induction	
First‐line	14 (77.8%)
Non‐first‐line	4 (22.2%)
Prior treatment (lines)	
Median (range)	1 (1–4)
Prior VEN‐based therapy	
Yes	1 (5.6%)
No	17 (94.4%)
Prior allo‐HSCT	
Yes	0 (0%)
No	18 (100%)
Therapeutic response of V‐HAG re‐induction	
CR	16 (88.9%)
CRi	2 (11.1%)
CRh	0 (0%)
FCM MRD after V‐HAG re‐induction	
Negative	16 (88.9%)
Positive	2 (11.1%)
Consolidation therapy	
V‐HAG only	8 (44.4%)
Allo‐HSCT	10 (55.6%)
Relapse	6 (33.3%)
V‐HAG only	6 (6/8, 75.0%)
Allo‐HSCT	0 (0/10, 0%)
Death	6 (33.3%)
PD	5 (5/6, 83.3%)
Others	1 (1/6, 16.7%)

Abbreviations: Allo‐HSCT, allogeneic hematopoietic stem cell transplantation; CR, complete remission; CRh, CR with partial hematological recovery; CRi, CR with incomplete hematological recovery; FCM, flow cytometry; MRD, minimal residual disease; PD, progressive disease.

**TABLE 2:** Genetic alterations in ETP‐ALL patients (*n* = 18).

Genetic alteration	Count (percentage)
Mutation (*N* ≥ 2)	
*DNMT3A*	6 (33.3%)
*KRAS*	5 (27.8%)
*NOTCH1*	5 (27.8%)
*PHF6*	4 (22.2%)
*ASXL1*	3 (16.7%)
*NRAS*	3 (16.7%)
*IDH2*	3 (16.7%)
*IKZF1*	3 (16.7%)
*TP53*	2 (11.1%)
*NF1*	2 (11.1%)
*JAK1*	2 (11.1%)
*JAK3*	2 (11.1%)
*RUNX1*	2 (11.1%)
*SETD2*	2 (11.1%)
Fusion	
*PICALM::MLLT10*	2 (11.1%)

### 2.2 Efficacy

#### 2.2.1 Response

Following one cycle of re‐induction with the V‐HAG regimen, the cCR rate reached 100% (18/18), encompassing 16 patients with CR and 2 patients with CR with incomplete hematological recovery (CRi). Minimal residual disease (MRD) was monitored via flow cytometry (FCM), and 16 patients achieved MRD negativity. The two patients who initially tested MRD‐positive also converted to MRD‐negative status after receiving an additional cycle of the V‐HAG regimen as consolidation therapy. No mortality events occurred during the V‐HAG re‐induction period (Table 1).

#### 2.2.2 Survival

**FIGURE 2:**
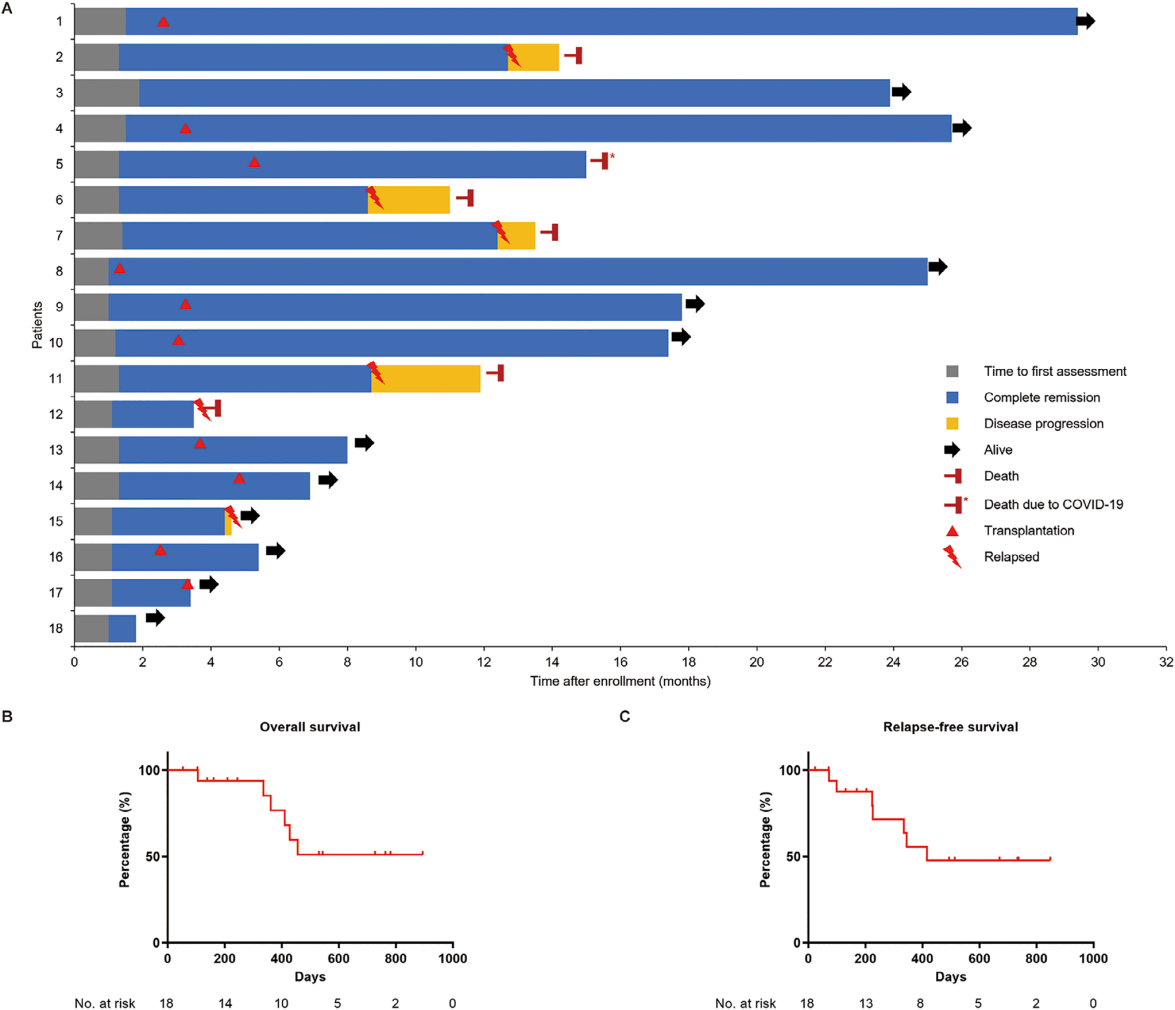
**Clinical outcomes of patients with refractory/relapsed ETP‐ALL**. (**A**) Therapeutic responses to the V‐HAG regimen were evaluated in refractory/relapsed ETP‐ALL patients. (**B, C**) Survival analysis was performed in these patients following re‐induction therapy with the V‐HAG regimen, with overall survival (**B**) and relapse‐free survival (**C**) presented.

**FIGURE 3:**
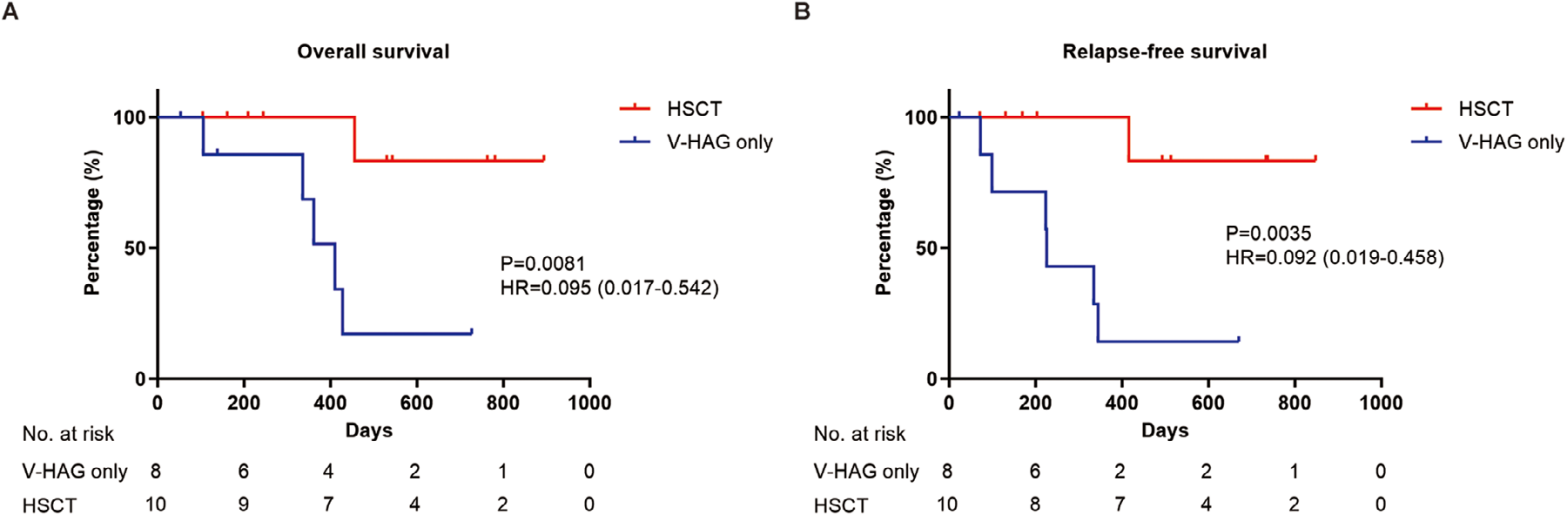
**Subgroup analysis of prognosis in refractory/relapsed ETP‐ALL patients who received HSCT or did not**. (**A, B**) Overall survival (**A**) and relapse‐free survival (**B**) of refractory/relapsed ETP‐ALL patients treated with the V‐HAG regimen were stratified by receipt of HSCT or not.

After achieving CR, 8 patients received continuous consolidation therapy with the V‐HAG regimen (V‐HAG only group), while 10 patients were bridged to allo‐HSCT (HSCT group). During a median follow‐up duration of 12.9 months (range: 1.8–29.8 months), 12 patients maintained CR at the last follow‐up, whereas 6 patients experienced disease relapse. In the V‐HAG only group, only 2 patients remained in CR at the data cut‐off date. In contrast, no patients in the HSCT group were found to have relapsed. Among the study cohort, 5 patients died due to progressive disease (PD), and 1 patient died of COVID‐19 infection following HSCT (Figure 2A; Table 1). The median overall survival (OS) and relapse‐free survival (RFS) durations were not reached. The 1‐year OS and 1‐year RFS rates were 76.7% (95% confidence interval [CI]: 53.2%–100.0%) and 55.7% (95% CI: 28.8%–82.6%), respectively (Figure 2B,C). Though all patients achieved CR after V‐HAG re‐induction, subgroup analysis revealed that, compared with the V‐HAG only group, the HSCT group exhibited significantly higher recurrent rate (75% vs. 0%, *p* = 0.0015) and mortality rate (62.5% vs. 10%, *p* = 0.0430) (Table S1). Consistently, a significant advantage in both OS (*p* = 0.0081; hazard ratio [HR] = 0.095, 95% CI: 0.017–0.542) and RFS (*p* = 0.0035; HR = 0.092, 95% CI: 0.019–0.458) was found in the HSCT group rather than the V‐HAG only group (Figure 3A,B).

### 2.3 Safety

As indicated, all patients who received the V‐HAG regimen experienced chemotherapy‐induced bone marrow suppression; therefore, hematological adverse events (AEs) were the most common. Neutropenia, anemia, and thrombocytopenia occurred in 100% of patients, and nearly all these hematological AEs were of grade 3 or higher, except for two patients who had grade 1 or 2 anemia. The median durations of neutropenia (neutrophil count < 0.5 × 10⁹/L), anemia (hemoglobin level <60 g/L), and thrombocytopenia (platelet count < 20 × 10⁹/L) were 10 (range: 0–33), 8 (range: 0–29), and 9 (range: 0–33) days, respectively. Regarding nonhematological AEs, infection (61.1%), liver injury (38.9%), and bleeding (33.3%) were prevalent, affecting more than one‐third of the patients. Notably, grade 3 or 4 nonhematological AEs were rare: only two patients (11.1%) experienced grade 3 or 4 infection, and one patient (5.6%) experienced grade 3 or 4 pneumonia (Table 3). No patients died during V‐HAG induction. Collectively, the V‐HAG regimen demonstrated a favorable safety profile as a re‐induction therapy for patients with refractory/relapsed ETP‐ALL.

**TABLE 3:** Adverse events (AE) of V‐HAG reinduction for refractory/relapsed ETP‐ALL.

Event	Any grade, *N* (%)	Grade≥3, *N* (%)
Hematological AEs		
Neutropenia	18 (100%)	18 (100%)
Anemia	18 (100%)	16 (88.9%)
Thrombocytopenia	18 (100%)	18 (100%)
Nonhematological AEs		
Bleeding	6 (33.3%)	0 (0%)
Infection	11 (61.1%)	2 (11.1%)
Pneumonia	4 (22.2%)	1 (5.6%)
Nausea, vomiting and diarrhea	5 (27.8%)	0 (0%)
Liver damage	7 (38.9%)	0 (0%)
Kidney damage	0 (0%)	0 (0%)
Tumor lysis syndrome	1 (5.6%)	0 (0%)
Drug fever	2 (11.1%)	0 (0%)

## 3 Discussion

In this phase 2 clinical study, we demonstrated that the V‐HAG regimen achieved a 100% cCR rate in patients with refractory/relapsed ETP‐ALL. Furthermore, these patients were more likely to maintain long‐term remission when followed by allo‐HSCT rather than continuous V‐HAG consolidation therapy. Thus, V‐HAG has been established as a crucial bridging regimen to allo‐HSCT for patients with refractory/relapsed ETP‐ALL.

As outlined in current clinical guidelines, intensive chemotherapy remains the standard treatment for both newly diagnosed and refractory/relapsed ETP‐ALL. Nelarabine‐based chemotherapy is the most widely used salvage therapy for refractory/relapsed ETP‐ALL; however, it only yields a CR rate of 36%–46%, highlighting an urgent need for alternative therapeutic strategies [6, 7, 8]. Although a considerable number of targeted agents have shown promising potential in ETP‐ALL treatment, their clinical role remains unestablished due to the lack of expert evaluation through well‐designed clinical trials. Given the high dependence of ETP‐ALL on BCL2, VEN‐based therapies have been extensively developed and applied in the treatment of refractory/relapsed ETP‐ALL. As previously reported, VEN has been combined with a variety of agents in such regimens, including the nucleoside analogues nelarabine or cladribine, the CD38 monoclonal antibody daratumumab, the proteasome inhibitor bortezomib, the HMAs decitabine or azacitidine, and chemotherapy regimens (e.g., CAG, HAG, HAAG, CAGE, Hyper‐CVAD/MA, or Mini‐CVD/MA) [17, 31, 32, 33, 34, 35, 36, 37, 38, 39, 40, 41, 42, 43]. This diversity of combinations reflects the role of VEN as the backbone of these therapeutic regimens. Consistent with this, several successful treatment cases have been reported; however, most of these studies are limited to retrospective case reports or case series. Therefore, the actual response rate of VEN‐based therapy in refractory/relapsed ETP‐ALL still requires further investigation. Our preclinical and pilot clinical studies have shown that HHT exerts a synergistic effect with VEN in ETP‐ALL. Moreover, when VEN is combined with the HAG regimen, a remarkable response was observed in patients with refractory/relapsed ETP‐ALL. On the basis of these findings, we investigated V‐HAG as a re‐induction therapy for patients with refractory/relapsed ETP‐ALL in our phase 2 trial, which resulted in an impressive 100% cCR rate.

ETP‐ALL is a stem cell‐like leukemia characterized by poor differentiation; thus, BCL2 dependence represents a universal and hallmark feature of this disease [1, 16]. Consistently, ETP‐ALL exhibits high sensitivity to VEN; however, upregulation of MCL‐1 and its binding to released BIM following VEN exposure may mediate VEN resistance [44]. It has been demonstrated that VEN exerts a synergistic effect with HHT or cytarabine in the treatment of ETP‐ALL [28, 45]. Mechanistically, both HHT and cytarabine induce MCL‐1 down‐regulation through caspase activation and proteasomal degradation [28, 44]. Furthermore, G‐CSF can stimulate quiescent G0‐phase cells to enter the G1 phase and mobilize bone marrow leukemic cells to the peripheral blood, thereby sensitizing ETP‐ALL cells to therapeutic agents [46]. As indicated, in the V‐HAG regimen, VEN plays a central role in mediating the antileukemic effect against ETP‐ALL. In addition, HHT and cytarabine prevent VEN resistance by promoting MCL‐1 degradation, while G‐CSF forces leukemic cells to exit the quiescent state and detach from the bone marrow microenvironment, both of which enhance drug sensitivity.

In addition, two studies involving a substantial number of VEN‐treated ETP‐ALL patients have been reported. In the prospective phase 1 trial by Hui Shi et al. [32], 21 patients with refractory/relapsed T‐ALL/lymphoblastic lymphoma (LBL) received the VEN plus daratumumab and CAGE regimen as re‐induction therapy; among these patients, 7 exhibited an ETP phenotype. Notably, T‐ALL/LBL patients with the ETP phenotype demonstrated a significantly superior response compared with those with a non‐ETP phenotype. For ETP T‐ALL/LBL patients, a bone marrow CR rate of 100% (6/6) and an extramedullary CR rate of 66.7% (2/3) were achieved. As highlighted in this study, ETP‐ALL, rather than non‐ETP ALL, was more sensitive to VEN‐based therapy, which reflects its reliance on BCL2. Consistent with our findings, nearly all ETP‐ALL patients in this study responded to VEN‐based therapy. However, the results of VEN‐based therapy have not been uniformly favorable across all studies. In the retrospective study by Jinyu Kong et al. [17], 15 patients with refractory/relapsed ETP‐ALL received VEN‐based therapy: 8 patients were treated with VEN plus HMA, 4 with VEN plus HMA and HAAG, 2 with VEN plus HMA and CAG, and 1 with VEN plus cladribine. The overall CR rate was 66.7% (10/15), which was significantly lower than that observed in our study. Subgroup analysis showed that the CR rate was 75.0% (6/8) in the chemo‐free group (VEN plus HMA) and 57.1% (4/7) in the chemo‐containing group (all other combinations). These findings indicate that ETP‐ALL patients exhibit varying responses to different VEN‐based regimens. Although VEN plays a central role in ETP‐ALL treatment, the choice of its combination partner is also a critical determinant of treatment response. As our data suggest, the HAG regimen appears to be an ideal partner for VEN in this setting.

CD7‐directed chimeric antigen receptor T‐cell (CAR‐T) therapy is an emerging treatment option for refractory/relapsed T‐ALL/LBL, with CR rates exceeding 80% and even reaching 95% [47, 48]. Compared with CAR‐T therapy, the V‐HAG regimen is significantly more accessible to most patients and has demonstrated a superior response in patients with ETP‐ALL. Thus, V‐HAG may serve as the first‐line salvage therapy for patients with refractory/relapsed ETP‐ALL, while CD7‐directed CAR‐T therapy could be considered a supplementary treatment for those who develop resistance to the V‐HAG regimen [49].

Allo‐HSCT remains the only therapeutic modality capable of maintaining long‐term CR and potentially achieving a cure for ETP‐ALL. The V‐HAG regimen acts as a bridging therapy, creating opportunities for patients to undergo allo‐HSCT while in CR [50]. In our study, V‐HAG was administered continuously until allo‐HSCT became available. Unexpectedly, most patients in the chemotherapy‐only consolidation group lost CR, whereas all patients in the allo‐HSCT group maintained CR, resulting in significant advantages in OS and RFS for patients who received allo‐HSCT. This outcome may be attributed to the high aggressiveness of ETP‐ALL, which renders the disease prone to developing resistance to repeated therapies (such as V‐HAG) within a relatively short timeframe. Therefore, we recommend that allo‐HSCT be performed as soon as patients with refractory/relapsed ETP‐ALL achieve CR via V‐HAG. Our conclusion is further supported by the study by Jinyu Kong et al. [17], in which 88.9% (8/9) of patients who achieved CR underwent allo‐HSCT, and 75% (6/8) of these patients remained relapse‐free at the last follow‐up. Allo‐HSCT also significantly prolonged OS in this cohort. Interestingly, the study by Hui Shi et al. [32] demonstrated that both allo‐HSCT and CAR‐T therapy have the potential to maintain long‐term CR in patients with refractory/relapsed ETP‐ALL following VEN‐based therapy—providing additional options for consolidation strategies.

Given the striking response of ETP‐ALL to VEN‐based therapy, several questions require further investigation in future studies. First, compared with newly diagnosed patients, most patients with refractory/relapsed ETP‐ALL have undergone multiple cycles of chemotherapy, and their tolerance to additional chemotherapy may be relatively poor. Thus, it remains to be determined whether a similar response can be achieved when the duration of VEN exposure is reduced to 14 days. Second, under current therapeutic strategies, the induction CR rate for newly diagnosed ETP‐ALL is approximately 90%–95%; therefore, it is unclear whether the V‐HAG regimen can achieve efficacy comparable to that of standard induction therapy (our ongoing trials: ChiCTR2200061708, NCT06361329). Third, chemo‐free regimens have demonstrated favorable safety profiles and promising efficacy in ETP‐ALL, raising the question of whether optimizing VEN's combination partner to targeted agents is feasible. Fourth, whether prior therapeutic history or genetic background influences the efficacy of the V‐HAG regimen needs to be validated in a larger cohort of ETP‐ALL patients treated with this regimen.

This study also has several limitations. First, only 18 evaluable patients were included, and the efficacy of the V‐HAG regimen requires further verification through additional clinical practice. Second, the V‐HAG regimen simply combines VEN with the existing HAG regimen; thus, optimization of the dosage and exposure duration of each component may be necessary for ETP‐ALL patients. Third, novel available therapies, such as CAR‐T therapy and additional targeted agents, could be integrated into consolidation strategies to further improve OS and RFS.

In summary, the V‐HAG regimen demonstrates remarkable efficacy in the treatment of refractory/relapsed ETP‐ALL and has been established as a critical bridging therapy to allo‐HSCT for patients with this disease.

## 4 Materials and Methods

### 4.1 Study Design and Patients

This is a prospective, multicenter, single‐arm, open‐label phase 2 clinical trial. The study was registered in the Chinese Clinical Trial Register (identifier: ChiCTR2100048966). It was conducted in accordance with the Declaration of Helsinki and was approved by the Institutional Review Board of the First Affiliated Hospital of Zhejiang University School of Medicine (approval number: IIT20210051C‐R1). Written informed consent was obtained from all patients before the initiation of any study‐related procedures.

Patients were enrolled in this study if they met the following eligibility criteria: (1) fulfilled the immunophenotypic criteria for ETP‐ALL (Table S2) [51, 52]; (2) had relapsed or refractory disease, where “relapsed” was defined as the re‐emergence of blasts in the bone marrow or peripheral blood (>5%) or confirmed extramedullary involvement after achieving CR, and “refractory” was defined as failure to achieve CR, CRi, or CR with partial hematological recovery (CRh) following completion of induction therapy (typically a 4‐week regimen or Hyper‐CVAD protocol); (3) were male or female and ≥18 years of age; (4) had an Eastern Cooperative Oncology Group (ECOG) performance status score of ≤2 (indicating expected tolerance to chemotherapy). Patients were excluded if they met any of the following criteria: a history of additional malignancies within the past 5 years; hepatic or renal insufficiency; inability to tolerate chemotherapy due to poor performance status (ECOG score ≥3); human immunodeficiency virus (HIV) or hepatitis C virus (HCV) infection; or pregnancy.

In refractory/relapsed ETP‐ALL, the CR rate of currently available therapies is approximately 36%–46%. Therefore, for sample size calculation, the hypothesized CR rate of standard treatment (null hypothesis, P_0_) was set at 0.40, while the expected CR rate of the V‐HAG regimen (alternative hypothesis, P_1_) was set at 0.85 based on the absence of V‐HAG re‐induction failure in ETP‐ALL patients in our previous pilot study. Assuming a two‐sided α (type I error) of 0.05 and a statistical power of 0.80, the study required at least 8 evaluable patients. Considering a potential 15% dropout rate, the minimum enrollment target for this study was set at 10 participants.

### 4.2 Treatment Procedures and Response Assessment

All patients with relapsed/refractory ETP‐ALL received the V‐HAG regimen, consisting of VEN, HHT, cytarabine, and G‐CSF, as re‐induction chemotherapy. The specific administration protocol was as follows: VEN: 100 mg orally on day 1, 200 mg orally on day 2, and 400 mg orally once daily from day 3 to day 28; HHT: 1.4 mg/m^2^ intravenously once daily from day 1 to day 10 (total daily dosage not exceeding 2 mg); Cytarabine: 10 mg/m^2^ subcutaneously every 12 h from day 1 to day 14; G‐CSF: 200 µg/m^2^ subcutaneously once daily from day 1 to day 14 (administration was discontinued when the white blood cell [WBC] count exceeded 20 × 10⁹/L). The dosage of VEN is modified in accordance with the following principles: (1) drug–drug interactions with CYP3A modulators: During the VEN dose escalation period, concurrent use of potent CYP3A inhibitors is prohibited. During the maintenance treatment period: When combined with a moderate CYP3A inhibitor, the VEN dose is reduced by 50%; When combined with a potent CYP3A inhibitor, the VEN dose is reduced by 75%; When combined with a potent CYP3A inducer, the VEN dose is increased to 600 mg per day. (2) Renal function abnormalities: Patients with mild, moderate, or severe renal function impairment (creatinine clearance rate ≥ 15 mL/min) do not require VEN dosage adjustment. (3) Liver function abnormalities: Patients with mild (Child–Pugh class A) or moderate (Child–Pugh class B) liver function impairment do not require VEN dosage adjustment; For patients with severe (Child–Pugh class C) liver function impairment, the daily VEN dose is reduced by 50%.

Disease response evaluations were performed within one week (days 29–35) following the first cycle of V‐HAG therapy. Therapeutic responses included CR, CRi, CRh, refractory disease, and PD. The assessment criteria for therapeutic responses were referenced from the Chinese Guideline for Adult Acute Lymphoblastic Leukemia and the NCCN Guideline for Adult Acute Lymphoblastic Leukemia. Patients who achieved CR/CRi/CRh at the end of the first cycle continued V‐HAG treatment or were bridged to allo‐HSCT for consolidation therapy. In contrast, patients who did not achieve CR/CRi/CRh at the end of the first cycle discontinued V‐HAG treatment. All patients received the study treatment until the occurrence of treatment failure (i.e., disease relapse), intolerable toxicity, death, or withdrawal of consent. The following situations should also be considered for treatment interruption: (1) During the dose escalation period of VEN, if tumor lysis syndrome (TLS) or TLS‐related biochemical abnormalities occur, VEN dose escalation must be halted, or VEN administration suspended. (2) Throughout the entire treatment course, if life‐threatening infections, life‐threatening bleeding, or severe acute liver or kidney dysfunction develop, administration of the V‐HAG regimen (or VEN, as clinically applicable) should be discontinued immediately. Additional therapeutic interventions were individualized based on each patient's response and clinical assessments. Prophylactic supportive care strategies mainly included: (1) Hematological adverse event management: G‐CSF was administered for the management of neutropenia. Red blood cell transfusions were used to treat anemia, while thrombocytopenia was managed with platelet transfusions and thrombopoietin receptor agonists. (2) Infection control: Infections were treated with antimicrobial agents, including antibacterial, antiviral, or antifungal medications, as clinically indicated. (3) TLS prevention and management: TLS was prevented and treated through appropriate hydration, urinary alkalization, and diuresis. (4) Drug‐induced fever management: Drug‐induced fever was addressed with glucocorticoids or nonsteroidal anti‐inflammatory drugs (NSAIDs). (5) Organ toxicity management: Liver damage and kidney damage were primarily treated with targeted pharmacotherapies. (6) Gastrointestinal symptom management: Nausea, vomiting, and diarrhea were managed with symptomatic treatment tailored to each patient's presentation.

### 4.3 Study Endpoints

The primary endpoint was cCR, defined as the proportion of patients achieving CR, CRi, or CRh at the end of V‐HAG re‐induction therapy. Secondary endpoints included OS, RFS, and safety. OS was calculated from the date of initiating V‐HAG treatment to the date of death from any cause. RFS was calculated from the date of achieving CR with V‐HAG treatment to the date of relapse or death from any cause. AEs were evaluated and graded in accordance with the Common Terminology Criteria for Adverse Events (CTCAE), Version 5.0.

### 4.4 Statistical Analysis

Sample size calculation was performed using PASS software. Data analysis was conducted with GraphPad Prism and SPSS Statistics software. For categorical variables, results were presented as counts and percentages within each category. For continuous variables, the Shapiro–Wilk test was used to assess normality. Continuous variables with a nonnormal distribution were presented as median (minimum, maximum), whereas those with a normal distribution were presented as mean (standard deviation). Survival analysis was performed using the Kaplan–Meier method, and between‐group differences were evaluated using the log‐rank test. A two‐sided *p*‐value <0.05 was considered statistically significant.

## Author Contributions

Wenjuan Yu and Jie Jin designed the research. Hongsheng Zhou, Liping Mao, Yinjun Lou, Lijing Shen, Ying Lu, Zhenfang Liu, Xiuzhen Tong, Aiping Zhang, Tingbo Liu, Na Zhang, Xingnong Ye, Juying Wei, Meihong Luo, Shaoyuan Wang, Qingxian Bai, Jian Hou, and Qifa Liu managed these patients. Xiang Zhang and Wenjuan Yu collected data and displayed the analysis. Hongyan Tong provided advice for our study. Xiang Zhang wrote the manuscript. Wenjuan Yu and Jie Jin revised the manuscript. All authors approved the manuscript.

## Funding Information

This study was funded by the National Key Research and Development Program of China (2022YFC2502700, 2022YFC2502701) and the National Natural Science Foundation of China (82370151).

## Conflicts of Interest

The authors declare no conflicts of interest.

## Ethics Statement

This study was approved by the ethical review committees of the First Affiliated Hospital of Zhejiang University School of Medicine (IIT20210051C‐R1). All procedures performed in studies involving human participants were in accordance with the ethical standards of the institutional and/or national research committee and with the 1964 Helsinki Declaration and its later amendments or comparable ethical standards. Informed consent was obtained from all individual participants included in the study.

## Supporting information



Table S1. The baseline characteristics of the V‐HAG‐only and HSCT groups.
Table S2. Immunophenotype scoring system for diagnosis of ETP‐ALL.

## Data Availability

The datasets used and/or analyzed during the current study are available from the corresponding author upon reasonable request.

## References

[1] J. Zhang , L. Ding , L. Holmfeldt , et al., “The Genetic Basis of Early T‐Cell Precursor Acute Lymphoblastic Leukaemia,” Nature 481, no. 7380 (2012): 157–163. 22237106 10.1038/nature10725 PMC3267575

[2] P. Polonen , C. G. Mullighan , and D. T. Teachey , “Classification and Risk Stratification in T‐Lineage Acute Lymphoblastic Leukemia,” Blood 145, no. 14 (2025): 1464–1474. 39357057 10.1182/blood.2023022920 PMC12002191

[3] C. F. Sin and P. M. Man , “Early T‐Cell Precursor Acute Lymphoblastic Leukemia: Diagnosis, Updates in Molecular Pathogenesis, Management, and Novel Therapies,” Frontiers in oncology 11 (2021): 750789. 34912707 10.3389/fonc.2021.750789 PMC8666570

[4] F. Tarantini , C. Cumbo , L. Anelli , et al., “Inside the Biology of Early T‐Cell Precursor Acute Lymphoblastic Leukemia: The Perfect Trick,” Biomarker Research 9, no. 1 (2021): 89. 34930475 10.1186/s40364-021-00347-z PMC8686563

[5] Y. Abaza , H. MK , S. Faderl , et al., “Hyper‐CVAD plus Nelarabine in Newly Diagnosed Adult T‐Cell Acute Lymphoblastic Leukemia and T‐Lymphoblastic Lymphoma,” American Journal of Hematology 93, no. 1 (2018): 91–99. 29047158 10.1002/ajh.24947

[6] A. Candoni , D. Lazzarotto , F. Ferrara , et al., “Nelarabine as Salvage Therapy and Bridge to Allogeneic Stem Cell Transplant in 118 Adult Patients With Relapsed/Refractory T‐Cell Acute Lymphoblastic Leukemia/Lymphoma. A CAMPUS ALL Study,” American Journal of Hematology 95, no. 12 (2020): 1466–1472. 32777149 10.1002/ajh.25957

[7] M. Kathpalia , P. Mishra , R. Bajpai , D. Bhurani , and N. Agarwal , “Efficacy and Safety of Nelarabine in Patients With Relapsed or Refractory T‐Cell Acute Lymphoblastic Leukemia: A Systematic Review and Meta‐Analysis,” Annal of Hematology 101, no. 8 (2022): 1655–1666. 10.1007/s00277-022-04880-1 35727338

[8] B. Samra , A. S. Alotaibi , N. J. Short , et al., “Outcome of Adults with Relapsed/Refractory T‐Cell Acute Lymphoblastic Leukemia or Lymphoblastic Lymphoma,” American Journal of Hematology 95, no. 9 (2020): E245–E247. 32501545 10.1002/ajh.25896

[9] Y. Zhang , J. J. Qian , Y. L. Zhou , et al., “Comparison of Early T‐Cell Precursor and Non‐ETP Subtypes among 122 Chinese Adults with Acute Lymphoblastic Leukemia,” Frontiers in Oncology 10 (2020): 1423. 32974153 10.3389/fonc.2020.01423 PMC7473208

[10] M. Neumann , S. Heesch , C. Schlee , et al., “Whole‐Exome Sequencing in Adult ETP‐ALL Reveals a High Rate of DNMT3A Mutations,” Blood 121, no. 23 (2013): 4749–4752. 23603912 10.1182/blood-2012-11-465138

[11] A. Bataller , M. Garrote , A. Oliver‐Caldes , et al., “Early T‐Cell Precursor Lymphoblastic Leukaemia: Response to FLAG‐IDA and High‐Dose Cytarabine With Sorafenib After Initial Refractoriness,” British Journal of Haematology 185, no. 4 (2019): 755–757. 30334573 10.1111/bjh.15601

[12] X. Zhang , J. Li , J. Jin , and W. Yu , “Relapsed/Refractory Early T‐Cell Precursor Acute Lymphoblastic Leukemia Was Salvaged by Venetoclax plus HAG Regimen,” Annal of Hematology 99, no. 2 (2020): 395–397. 10.1007/s00277-019-03902-9 31879788

[13] Society of Blood Disease Translational Medicine of China Anti‐Cancer A, Lymphoma Group CSoHCMA, Hematology Committee of China Medical Women's A . [Chinese Expert Consensus on Diagnosis and Treatment of Adult Early T Cell Precursor Acute Lymphoblastic Leukemia (2023)]. Zhonghua Xue Ye Xue Za Zhi = Zhonghua Xueyexue Zazhi 2023;44(12):977–982. 38503519 10.3760/cma.j.issn.0253-2727.2023.12.002 PMC10834867

[14] B. Eichhorst , C. U. Niemann , A. P. Kater , et al., “First‐Line Venetoclax Combinations in Chronic Lymphocytic Leukemia,” New England Journal of Medicine 388, no. 19 (2023): 1739–1754. 37163621 10.1056/NEJMoa2213093

[15] C. D. DiNardo , B. A. Jonas , V. Pullarkat , et al., “Azacitidine and Venetoclax in Previously Untreated Acute Myeloid Leukemia,” New England Journal of Medicine 383, no. 7 (2020): 617–629. 32786187 10.1056/NEJMoa2012971

[16] T. N. Chonghaile , J. E. Roderick , C. Glenfield , et al., “Maturation Stage of T‐Cell Acute Lymphoblastic Leukemia Determines BCL‐2 versus BCL‐XL Dependence and Sensitivity to ABT‐199,” Cancer discovery 4, no. 9 (2014): 1074–1087. 24994123 10.1158/2159-8290.CD-14-0353 PMC4154982

[17] J. Y. Kong , L. H. Zong , Y. Pu , et al., “Clinical Efficacy and Safety of Venetoclax Combined With Multidrug Chemotherapy in the Treatment of 15 Patients with Relapsed or Refractory Early T‐Cell Precursor Acute Lymphoblastic Leukemia,” Zhonghua Xue Ye Xue Za Zhi = Zhonghua Xueyexue Zazhi 44, no. 8 (2023): 649–653. 37803838 10.3760/cma.j.issn.0253-2727.2023.08.006 PMC10520236

[18] V. Agrawal , S. Arslan , H. Pourhassan , P. Koller , I. Aldoss , and V. Pullarkat , “Hypomethylating Agent and Venetoclax Are Effective Salvage Therapies in Relapsed/Refractory Early T‐Cell Precursor Acute Lymphoblastic Leukaemia,” British Journal of Haematology 206, no. 6 (2025): 1678–1682. 40205647 10.1111/bjh.20065

[19] S. S. Lam , E. S. Ho , B. L. He , et al., “Homoharringtonine (omacetaxine mepesuccinate) as an Adjunct for FLT3‐ITD Acute Myeloid Leukemia,” Science Translational Medicine 8, no. 359 (2016): 359ra129. 10.1126/scitranslmed.aaf3735 27708062

[20] J. Jin , J. X. Wang , F. F. Chen , et al., “Homoharringtonine‐based Induction Regimens for Patients With De‐novo Acute Myeloid Leukaemia: A Multicentre, Open‐Label, Randomised, Controlled Phase 3 Trial,” The Lancet Oncology 14, no. 7 (2013): 599–608. 23664707 10.1016/S1470-2045(13)70152-9

[21] C. Li , L. Dong , R. Su , et al., “Homoharringtonine Exhibits Potent Anti‐Tumor Effect and Modulates DNA Epigenome in Acute Myeloid Leukemia by Targeting SP1/TET1/5hmC,” Haematologica 105, no. 1 (2020): 148–160. 30975912 10.3324/haematol.2018.208835 PMC6939512

[22] S. Huang , J. Pan , J. Jin , et al., “Abivertinib, a Novel BTK Inhibitor: Anti‐Leukemia Effects and Synergistic Efficacy With Homoharringtonine in Acute Myeloid Leukemia,” Cancer Letters 461 (2019): 132–143. 31310800 10.1016/j.canlet.2019.07.008

[23] Q. Ling , F. Li , X. Zhang , et al., “MAP4K1 Functions as a Tumor Promotor and Drug Mediator for AML via Modulation of DNA Damage/Repair System and MAPK Pathway,” EBioMedicine 69 (2021): 103441. 34166980 10.1016/j.ebiom.2021.103441 PMC8239467

[24] E. K. Allan , T. L. Holyoake , A. R. Craig , and H. G. Jorgensen , “Omacetaxine May Have a Role in Chronic Myeloid Leukaemia Eradication Through Downregulation of Mcl‐1 and Induction of Apoptosis in Stem/Progenitor Cells,” Leukemia 25, no. 6 (2011): 985–994. 21468038 10.1038/leu.2011.55

[25] Q. Zhang , B. Riley‐Gillis , L. Han , et al., “Activation of RAS/MAPK Pathway Confers MCL‐1 Mediated Acquired Resistance to BCL‐2 Inhibitor Venetoclax in Acute Myeloid Leukemia,” Signal Transduct Target Ther 7, no. 1 (2022): 51. 35185150 10.1038/s41392-021-00870-3 PMC8858957

[26] H. Jin , Y. Zhang , S. Yu , et al., “Venetoclax Combined with Azacitidine and Homoharringtonine in Relapsed/Refractory AML: A Multicenter, Phase 2 Trial,” Journal of hematology & oncology 16, no. 1 (2023): 42. 37120593 10.1186/s13045-023-01437-1 PMC10149010

[27] S. Suo , D. Zhao , F. Li , et al., “Homoharringtonine Inhibits the NOTCH/MYC Pathway and Exhibits Antitumor Effects in T‐Cell Acute Lymphoblastic Leukemia,” Blood 144, no. 12 (2024): 1343–1347. 38968151 10.1182/blood.2023023400 PMC11451333

[28] S. Suo , S. Sun , L. X. T. Nguyen , et al., “Homoharringtonine Synergizes with Venetoclax in Early T Cell Progenitor Acute Lymphoblastic Leukemia: Bench and Bed,” Med 5, no. 12 (2024): 1510–1524. 39151422 10.1016/j.medj.2024.07.018

[29] Y. Shi , J. Ye , Y. Yang , et al., “The Basic Research of the Combinatorial Therapy of ABT‐199 and Homoharringtonine on Acute Myeloid Leukemia,” Frontiers in Oncology 11 (2021): 692497. 34336680 10.3389/fonc.2021.692497 PMC8317985

[30] W. Wei , S. Huang , Q. Ling , et al., “Homoharringtonine Is Synergistically Lethal with BCL‐2 Inhibitor APG‐2575 in Acute Myeloid Leukemia,” Journal of Translational Medicine 20, no. 1 (2022): 299. 35794605 10.1186/s12967-022-03497-2 PMC9258085

[31] S. Arora , P. Vachhani , K. Bachiashvili , and O. Jamy , “Venetoclax with Chemotherapy in Relapse/Refractory Early T‐Cell Precursor Acute Lymphoblastic Leukemia,” Leukemia & Lymphoma 62, no. 9 (2021): 2292–2294. 33691573 10.1080/10428194.2021.1897807

[32] H. Shi , F. Yang , M. Cao , et al., “Daratumumab and Venetoclax Combined With CAGE for Late R/R T‐ALL/LBL Patients: Single‐Arm, Open‐Label, Phase I Study,” Annal of Hematology 103, no. 8 (2024): 2993–3004. 10.1007/s00277-024-05775-z 38662205

[33] G. Richard‐Carpentier , E. Jabbour , N. J. Short , et al., “Clinical Experience with Venetoclax Combined with Chemotherapy for Relapsed or Refractory T‐Cell Acute Lymphoblastic Leukemia,” Clinical Lymphoma, Myeloma & Leukemia 20, no. 4 (2020): 212–218. 10.1016/j.clml.2019.09.608 32035785

[34] A. McEwan , O. Pitiyarachchi , and N. Viiala , “Relapsed/Refractory ETP‐ALL Successfully Treated with Venetoclax and Nelarabine as a Bridge to Allogeneic Stem Cell Transplant,” Hemasphere 4, no. 3 (2020): e379. 32647798 10.1097/HS9.0000000000000379 PMC7306301

[35] Y. Numan , M. Alfayez , A. Maiti , et al., “First Report of Clinical Response to Venetoclax in Early T‐Cell Precursor Acute Lymphoblastic Leukemia,” JCO Precision Oncology 2 (2018): PO1800127. 10.1200/PO.18.00127 PMC6508880 31080940

[36] W. Guan , Y. Jing , L. P. Dou , M. Q. Wang , Y. Xiao , and L. Yu , “Chidamide in Combination With Chemotherapy in Refractory and Relapsed T Lymphoblastic Lymphoma/Leukemia,” Leukemia Lymphoma 61, no. 4 (2020): 855–861. 31755348 10.1080/10428194.2019.1691195

[37] M. Neumann , E. Coskun , L. Fransecky , et al., “FLT3 Mutations in Early T‐Cell Precursor ALL Characterize a Stem Cell Like Leukemia and Imply the Clinical Use of Tyrosine Kinase Inhibitors,” PLoS ONE 8, no. 1 (2013): e53190. 23359050 10.1371/journal.pone.0053190 PMC3554732

[38] Y. Chen , L. Zhang , J. K. Huang , et al., “Dasatinib and Chemotherapy in a Patient With Early T‐Cell Precursor Acute Lymphoblastic Leukemia and *NUP214‐ABL1* Fusion: A Case Report,” Exp Ther Med 14, no. 5 (2017): 3979–3984. 29067094 10.3892/etm.2017.5046 PMC5647690

[39] S. L. Maude , S. Dolai , C. Delgado‐Martin , et al., “Efficacy of JAK/STAT Pathway Inhibition in Murine Xenograft Models of Early T‐Cell Precursor (ETP) Acute Lymphoblastic Leukemia,” Blood 125, no. 11 (2015): 1759–1767. 25645356 10.1182/blood-2014-06-580480 PMC4357583

[40] Y. X. Jiang , L. Ji , and X. Jin , “Case Report: Treatment of Two Cases of Recurrent/Refractory Early T‐Cell Precursor Acute Lymphoblastic Leukemia With Venetoclax Combined With the CAG Regimen,” Front Med‐Lausanne 11 (2024): 1358161. 38523911 10.3389/fmed.2024.1358161 PMC10957540

[41] M. Espinoza , P. Ramirez , Y. Najima , and J. Rojas‐Vallejos , “Successful Stem Cell Transplantation After Nelarabine, Pegylated Asparaginase, Vincristine, Doxorubicin, and Prednisone in Refractory Early T‐Cell Precursor Acute Lymphoblastic Leukemia: A Case Report,” Medwave 23, no. 4 (2023): 2664.

[42] J. Y. Kong , N. Chen , M. Y. Li , et al., “Venetoclax and Decitabine in Refractory TP53‐Mutated Early T‐Cell Precursor Acute Lymphoblastic Leukemia,” Annals of Hematology 101, no. 3 (2022): 697–699. 33954816 10.1007/s00277-021-04530-y

[43] T. Y. Meng , Y. Yao , Y. Xu , et al., “Salvage Therapy With decitabine in Combination With Granulocyte Colony‐Stimulating Factor, Low‐Dose Cytarabine, and Aclarubicin in Patients With Refractory or Relapsed Early T‐Cell Precursor Acute Lymphoblastic Leukemia,” Hematological Oncology 38, no. 5 (2020): 834–837. 32710795 10.1002/hon.2783

[44] X. Niu , J. Zhao , J. Ma , et al., “Binding of Released Bim to Mcl‐1 Is a Mechanism of Intrinsic Resistance to ABT‐199 Which Can be Overcome by Combination With Daunorubicin or Cytarabine in AML Cells,” Clinical Cancer Research 22, no. 17 (2016): 4440–4451. 27103402 10.1158/1078-0432.CCR-15-3057 PMC5010519

[45] N. M. Anderson , I. Harrold , M. R. Mansour , et al., “BCL2‐Specific Inhibitor ABT‐199 Synergizes Strongly With Cytarabine Against the Early Immature LOUCY Cell Line but Not More‐differentiated T‐ALL Cell Lines,” Leukemia 28, no. 5 (2014): 1145–1148. 24342948 10.1038/leu.2013.377 PMC4013222

[46] A. Chen , J. Yang , S. Hu , and Q. F. Wang , “The Priming Induction Regimen of HAG as a Low Dose Chemotherapy Strategy in AML Clonal Evolution,” Sci China Life Sci 58, no. 12 (2015): 1302–1305. 26588914 10.1007/s11427-015-4974-5

[47] P. Lu , Y. Liu , J. Yang , et al., “Naturally Selected CD7 CAR‐T Therapy Without Genetic Manipulations for T‐ALL/LBL: First‐in‐Human Phase 1 Clinical Trial,” Blood 140, no. 4 (2022): 321–334. 35500125 10.1182/blood.2021014498

[48] Y. Tan , L. Shan , L. Zhao , et al., “Long‐term Follow‐up of Donor‐derived CD7 CAR T‐Cell Therapy in Patients with T‐Cell Acute Lymphoblastic Leukemia,” Journal of hematology & oncology 16, no. 1 (2023): 34. 37020231 10.1186/s13045-023-01427-3 PMC10074659

[49] H. P. Dai , W. Cui , Q. Y. Cui , et al., “Haploidentical CD7 CAR T‐Cells Induced Remission in a Patient with TP53 Mutated Relapsed and Refractory Early T‐Cell Precursor Lymphoblastic Leukemia/Lymphoma,” Biomarker Research 10, no. 1 (2022): 6. 35130959 10.1186/s40364-022-00352-w PMC8822664

[50] A. Schrauder , A. Reiter , H. Gadner , et al., “Superiority of Allogeneic Hematopoietic Stem‐Cell Transplantation Compared With Chemotherapy Alone in High‐Risk Childhood T‐Cell Acute Lymphoblastic Leukemia: Results from ALL‐BFM 90 and 95,” Journal of Clinical Oncology 24, no. 36 (2006): 5742–5749. 17179108 10.1200/JCO.2006.06.2679

[51] H. Khogeer , H. Rahman , N. Jain , et al., “Early T Precursor Acute Lymphoblastic Leukaemia/Lymphoma Shows Differential Immunophenotypic Characteristics Including Frequent CD33 Expression and In Vitro Response to Targeted CD33 Therapy,” British Journal of Haematology 186, no. 4 (2019): 538–548. 31115909 10.1111/bjh.15960

[52] D. A. Arber , A. Orazi , R. Hasserjian , et al., “The 2016 Revision to the World Health Organization Classification of Myeloid Neoplasms and Acute Leukemia,” Blood 127, no. 20 (2016): 2391–2405. 27069254 10.1182/blood-2016-03-643544

