# Supplementary material for: Venetoclax and Homoharringtonine‐Based Therapy Exhibited a Striking Response in Refractory/Relapsed Early T‐Cell Precursor Acute Lymphoblastic Leukemia
Source: MedComm (2020). 2025 Dec 12;6(12):e70549. doi: 10.1002/mco2.70549 (PMC12701281; doi:10.1002/mco2.70549)
Supplement: Supplementary file 1 — Table S1. The baseline characteristics of the V‐HAG‐only and HSCT groups. Table S2. Immunophenotype scoring system for diagnosis of ETP‐ALL. [file MCO2-6-e70549-s001.docx]

**Supplementary Information**

**Venetoclax and Homoharringtonine-based therapy exhibited a striking response in refractory/relapsed early T-cell precursor acute lymphoblastic leukemia**

**Authors**

Xiang Zhang^1^#, Hongsheng Zhou^2^#, Liping Mao^1^, Yinjun Lou^1^, Lijing Shen^3^, Ying Lu^4^, Zhenfang Liu^5^, Xiuzhen Tong^6^, Aiping Zhang^7^, Tingbo Liu^8^, Na Zhang^9^, Xingnong Ye^1^, Juying Wei^1^, Meihong Luo^7^, Shaoyuan Wang^8^, Qingxian Bai^9^, Jian Hou^3^, Qifa Liu^2^, Hongyan Tong^1^, Jie Jin^1^*, Wenjuan Yu^1^*

**Affiliations**

1 Department of Hematology, The First Affiliated Hospital, Zhejiang University School of Medicine, Hangzhou 310003, Zhejiang, PR China; 2 Department of Hematology, Nanfang Hospital, Southern Medical University, Guangzhou 510515, Guangdong, PR China; 3 Departments of Hematology, Renji Hospital, Shanghai Jiao Tong University School of Medicine, Shanghai 200136, Shanghai, PR China; 4 Department of Hematology, The Affiliated People's Hospital of Ningbo University, Ningbo 315211, Zhejiang, PR China; 5 Department of Hematology, The First Affiliated Hospital of Guangxi Medical University, Nanning 530021, Guangxi, PR China; 6 Department of Hematology, First Affiliated Hospital of Sun Yat sen University, Guangzhou 510080, Guangdong, PR China; 7 Department of Hematology, Baoshan Hospital Shanghai University of Traditional Chinese Medicine, Shanghai 201999, Shanghai, PR China; 8 Department of Hematology, Fujian Medical University Union Hospital, Fuzhou, 350001, Fujian, PR China; 9 Department of Hematology, the First Affiliated Hospital of Air Force Military Medical University, Xi'an 710032, Shaanxi, PR China.

#, These authors contributed equally to this work.

***Corresponding authors**

Jie Jin M.D. (E-mail: jiej0503@zju.edu.cn); Wenjuan M.D. (E-mail: drwjyu1977@zju.edu.cn).

**Table S1. The baseline characteristics of V-HAG only and HSCT groups.**

| **Characteristic** | **V-HAG only group** | **HSCT group** | **P** |
| --- | --- | --- | --- |
| No. of patients (N) | 8 | 10 |  |
| Age (years) |  |  |  |
| Median (range) | 57 (32-73) | 35 (19-65) | 0.2848 |
| Gender |  |  |  |
| Male (N, %) | 7 (87.5%) | 5 (50%) | 0.1516 |
| Female (N, %) | 1 (12.5%) | 5 (50%) |  |
| Hematologic parameters |  |  |  |
| Median (range) |  |  |  |
| White blood cell count (10^9^/L) | 1.165 (0.41-7.21) | 3.275 (0.72-9.68) | 0.2031 |
| Hemoglobin level (g/L) | 72 (59-123) | 91 (50-135) | 0.4237 |
| Platelet count (10^9^/L) | 46.5 (31-196) | 121 (8-408) | 0.3281 |
| Bone marrow blast |  |  |  |
| Median (range) | 72.5% (65%-95%) | 37.25% (8%-72.5%) | 0.0034 |
| Complete remission (N, %) | 8 (100%) | 10 (100%) | / |
| Relapse (N, %) | 6 (75.0%) | 0 (0%) | 0.0015 |
| Death (N, %) | 5 (62.5%) | 1 (10%) | 0.0430 |

**Table S2. Immunophenotype scoring system for diagnosis of ETP-ALL.**

| **Marker** | **Positive** | **Negative** |
| --- | --- | --- |
| CD1a | -2 | 2 |
| sCD3 | -2 |  |
| CD5 | -2 | 2 |
| CD8 |  | 2 |
| CD10 |  | 1 |
| CD13 | 1 |  |
| CD33 | 1 |  |
| CD34 | 1 |  |
| CD117 | 1 |  |
| TdT |  | 1 |
| MPO | -3 |  |

ETP-ALL: total score ≥ 8; non-ETP T-ALL: total score < 8.
